# A novel, likely pathogenic variant in *UBTF*‐related neurodegeneration with brain atrophy is associated with a severe divergent neurodevelopmental phenotype

**DOI:** 10.1002/mgg3.2054

**Published:** 2022-09-15

**Authors:** Rory J. Tinker, Tiffany Guess, David C. Rinker, Jonathan H. Sheehan, Daniel Lubarsky, Binu Porath, Mackenzie Mosera, Ping Mayo, Emily Solem, Laura A. Lee, Asha Sharam, Jennifer Brault

**Affiliations:** ^1^ Division of Medical Genetics and Genomic Medicine Vanderbilt University Medical Center Nashville Tennessee USA; ^2^ Department of Pathology, Microbiology, and Immunology Vanderbilt University Medical Center Nashville Tennessee USA; ^3^ Department of Biological Sciences Vanderbilt University Nashville Tennessee USA; ^4^ Department of Internal Medicine, Division of Infectious Diseases Washington University School of Medicine St. Louis Missouri USA; ^5^ Department of Radiology Vanderbilt University Medical Center Nashville Tennessee USA

**Keywords:** CONDBA, *UBTF*, whole exome sequencing

## Abstract

**Background:**

A de novo, pathogenic, missense variant in *UBTF*, c.628G>A p.Glu210Lys, has been described as the cause of an emerging neurodegenerative disorder, Childhood‐Onset Neurodegeneration with Brain Atrophy (CONDBA). The p.Glu210Lys alteration yields a positively charged stretch of three lysine residues. Functional studies confirmed this change results in a stronger interaction with negatively charged DNA and gain‐of‐function activity when compared to the wild‐type sequence. The CONDBA phenotype reported in association with p.Glu210Lys consists of normal early‐neurodevelopment followed by progressive motor, cognitive, and behavioral regression in early‐to‐middle childhood.

**Methods and Results:**

The current proband presented at 9 months of age with baseline developmental delay and more extensive neuroradiological findings, including pontine hypoplasia, thalamic volume loss and signal abnormality, and hypomyelination. Like the recurrent CONDBA p.Glu210Lys variant, this novel variant, c.608A>G p.(Gln203Arg) lies within the highly conserved second HMG‐box homology domain and involves the replacement of the wild‐type residue with a positively charged residue, arginine. Computational structural modeling demonstrates that this amino acid substitution potentiates the interaction between *UBTF* and DNA, likely resulting in a gain‐of‐function effect for the *UBTF* protein, UBF.

**Conclusion:**

Here we present a new divergent phenotype associated with a novel, likely pathogenic, missense variant at a different position in the *UBTF* gene, c.608A>G p.(Gln203Arg).

## Graphical abstract

A de novo, pathogenic, missense variant in UBTF, c.628G>A p.Glu210Lys, has been described as the cause of an emerging neurodegenerative disorder, Childhood‐Onset Neurodegeneration with Brain Atrophy (CONDBA). The p.Glu210Lys alteration yields a positively charged stretch of three lysine residues. Here we present a new divergent phenotype associated with a novel, likely pathogenic, missense variant at a different position in the UBTF gene, c.608A>G p.Gln203Arg. Computational structural modeling demonstrates that this amino acid substitution potentiates the interaction between UBTF and DNA, likely resulting in a gain‐of‐function effect for the UBTF protein.

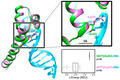

## 1 BACKGROUND

Whole‐exome sequencing (WES) has resulted in major advances in our understanding of the molecular etiology of human disease (100,000 Genomes Project Pilot Investigators, 2021; Splinter et al., 2018). This has led to a substantially improved diagnostic yield in a spectrum of genetic disorders ranging from metabolic to neurodevelopmental (Deciphering Developmental Disorders Study, 2017; Tinker et al., 2021). A recurrent, de novo pathogenic variant in *UBTF* (upstream binding transcription factor) (OMIM 600673), c.628G>A p.Glu210Lys (NM_014233.3), has been associated with childhood‐onset neurodegeneration with brain atrophy (CONDBA, OMIM 617672). CONDBA is characterized by a period of loss of motor and cognitive skills in the first decade of life following a period of normal development or mild/moderate developmental delay (Bastos et al., 2020; Edvardson et al., 2017; Ikeda et al., 2021; Sedláčková et al., 2019; Toro et al., 2018). This condition was first described in 2017 by Edvardson et al.(Tinker et al., 2021) and to date, only 14 patients have been reported in the literature (Supplementary Table S1). The median age of onset of neurodevelopmental regression is 3 years. Progressive cerebral and cerebellar atrophy results in loss of motor skills and language with profound intellectual disability in all patients with CONDBA (Bastos et al., 2020; Edvardson et al., 2017; Ikeda et al., 2021; Sedláčková et al., 2019; Toro et al., 2018). Mild developmental delay, microcephaly, ataxia, extrapyramidal and pyramidal signs, behavioral issues, dysarthria, dysphagia, epilepsy, abnormal EEG, and MRI abnormalities, including cerebellar and white matter atrophy, have been reported (Bastos et al., 2020; Edvardson et al., 2017; Ikeda et al., 2021; Sedláčková et al., 2019; Toro et al., 2018).

To date, the causative variant identified in all documented cases of CONDBA is a single heterozygous, de novo, missense gain‐of‐function alteration in *UBTF*, c.628G>A p.Glu210Lys (Bastos et al., 2020; Edvardson et al., 2017; Ikeda et al., 2021; Sedláčková et al., 2019; Toro et al., 2018), hereafter referred to as p.Glu210Lys. This recurrent variant occurs in exon 7 of *UBTF*, located in the highly conserved second high mobility group (HMG)‐box homology domain of UBF, the protein encoded by *UBTF*. UBF serves as one of the transcription factors for RNA polymerase I in mammals and plays a critical role in the generation of rRNA transcripts (Edvardson et al., 2017; Ikeda et al., 2021; Tinker et al., 2021). The p.Glu210Lys variant in *UBTF* results in a positively charged stretch of three lysine residues, which yields a stronger interaction with negatively charged DNA when compared to the wild‐type sequence (Tinker et al., 2021). In vitro functional studies using the cells from an affected individual with the p.Glu210Lys variant showed a significant increase in the expression of ribosomal subunit 18S. This result suggested that *UBTF* harboring the p.Glu210Lys variant causes the UBF protein to function as a hyperactive transcription factor leading to overexpression of rDNA (Tinker et al., 2021).

Here we report an 18‐month‐old female with severe early‐onset developmental delay and cerebral and cerebellar atrophy associated with a novel, presumed de novo, likely pathogenic variant (Richards et al., 2015) in *UBTF* c.608A>G p.(Gln203Arg) (NM_014233.3), hereafter referred to as p.(Gln203Arg), that is predicted to affect the HMG homology domain. Our proband has a presentation similar to patients diagnosed with CONDBA, however, with the addition of a severe early‐onset neurodevelopmental phenotype. Using computational structural modeling, we compared the energetic stability of wild‐type *UBTF* to *UBTF* p.(Gln203Arg) while in complex with DNA.

## 2 CASE REPORT

The proband's parents kindly provided informed consent for the publication of the current article. The proband was born to non‐consanguineous, Caucasian parents at term. Birth and the neonatal period were uncomplicated. The proband has no siblings. There is no reported family history of genetic or neurologic conditions. Global developmental delay was identified at 9 months of age by the proband's primary care physician. The patient was unable to sit with support, use crude grip, or babble.

On initial presentation to pediatric neurology at 11 months, the proband's phenotype consisted of failure to thrive (<1% for weight and length), feeding intolerance, microcephaly (<1%), and central hypotonia. A brain MRI demonstrated pontine hypoplasia.

At 12 months, the proband was evaluated by pediatric genetics. An initial evaluation consisting of a chromosomal microarray, Fragile X testing, plasma amino acid levels, urine organic acids, and an acylcarnitine profile was normal. At 15 months, WES of the proband was ordered.

At 18 months, in addition to unchanged pontine hypoplasia, a repeat MRI demonstrated progressive cerebral volume loss predominantly involving the white matter with mild callosal thinning, abnormal white matter signal consistent with hypomyelination (without appropriate progression of myelination compared to the examination at 12 months of age), symmetric volume loss and T2 prolongation of the thalami, and subtle cerebellar volume loss (Supplementary Figure S1). A routine, overnight EEG demonstrated frequent left frontal polar maximal sharp waves consistent with potential epileptogenicity, but no seizures were identified.

Results from the proband‐WES performed at 15 months of age identified five notable heterozygous variants of uncertain significance (VUSs) in *LRPPRC* (OMIN 607544), *POMK* (OMIN 615247), *WASHC4* (OMIN 615748), *SETD5* (OMIN 615743), and *UBTF* (Supplementary Table S1). While there was phenotypic overlap in our patient and syndromes associated with disease‐causing variants in *LRPPRC, POMK*, and *WASHC4*, pathogenic variants in these genes are rare and have only been associated with autosomal recessive disease (Di Costanzo et al., 2014; Oláhová et al., 2015; Ropers et al., 2011; von Renesse et al., 2014). Given this, we did not feel it necessary to perform parental testing for these variants. Pathogenic variants in *SETD5* and *UBTF*, however, are associated with autosomal dominant disease (Edvardson et al., 2017; Grozeva et al., 2014). This coupled with the phenotypic overlap of these genes' associated disorders and the proband's presentation compelled our Genomics Team to perform known familial variant testing via Sanger sequencing on both the *SETD5* and *UBTF* variants. Results from this testing showed the *SETD5* variant to be paternally inherited. The patient's parents are of normal intellectual and neurological ability; therefore, this variant was not believed to be a factor in the patient's condition.

Interestingly, the VUS in *UBTF* p.(Gln203Arg) was not identified in either parent, suggesting it is de novo in nature and allowing for the application of PM6 and the reclassification from VUS to likely pathogenic (Richards et al., 2015) (Supplementary Figure S2). Technical details on the sequencing approach are provided in the supplement.

This missense alteration in exon 7 of *UBTF* involves the the replacement of glutamine by arginine, a positively charged amino acid, at position 203 of the encoded protein. Similar to the recurrent p.Glu210Lys variant, p.(Gln203Arg) lies within the highly conserved second HMG‐box homology domain (100,000 Genomes Project Pilot Investigators, 2021) and involves replacement of the wild‐type residue with a positively charged amino acid, arginine. A variety of in silico algorithms predict a damaging effect of p.(Gln203Arg) on *UBTF* function (19 deleterious predictions; 5 benign predictions), and the variant has a REVEL score of 0.82 (Kopanos et al., 2019). Subsequent reevaluation of the patient by neurology identified developmental regression and repeat MRI results showed cerebral and cerebellar atrophy (Supplementary Figure S1). Noteworthy is the fact that our patient has several distinct phenotypic features in comparison to CONDBA patients described previously, including developmental delay and multiple neuroimaging findings including pontine hypoplasia, thalamic volume loss and signal abnormality, and hypomyelination, suggesting a more severe phenotype associated with the p.(Gln203Arg) variant.

**FIGURE 1:**
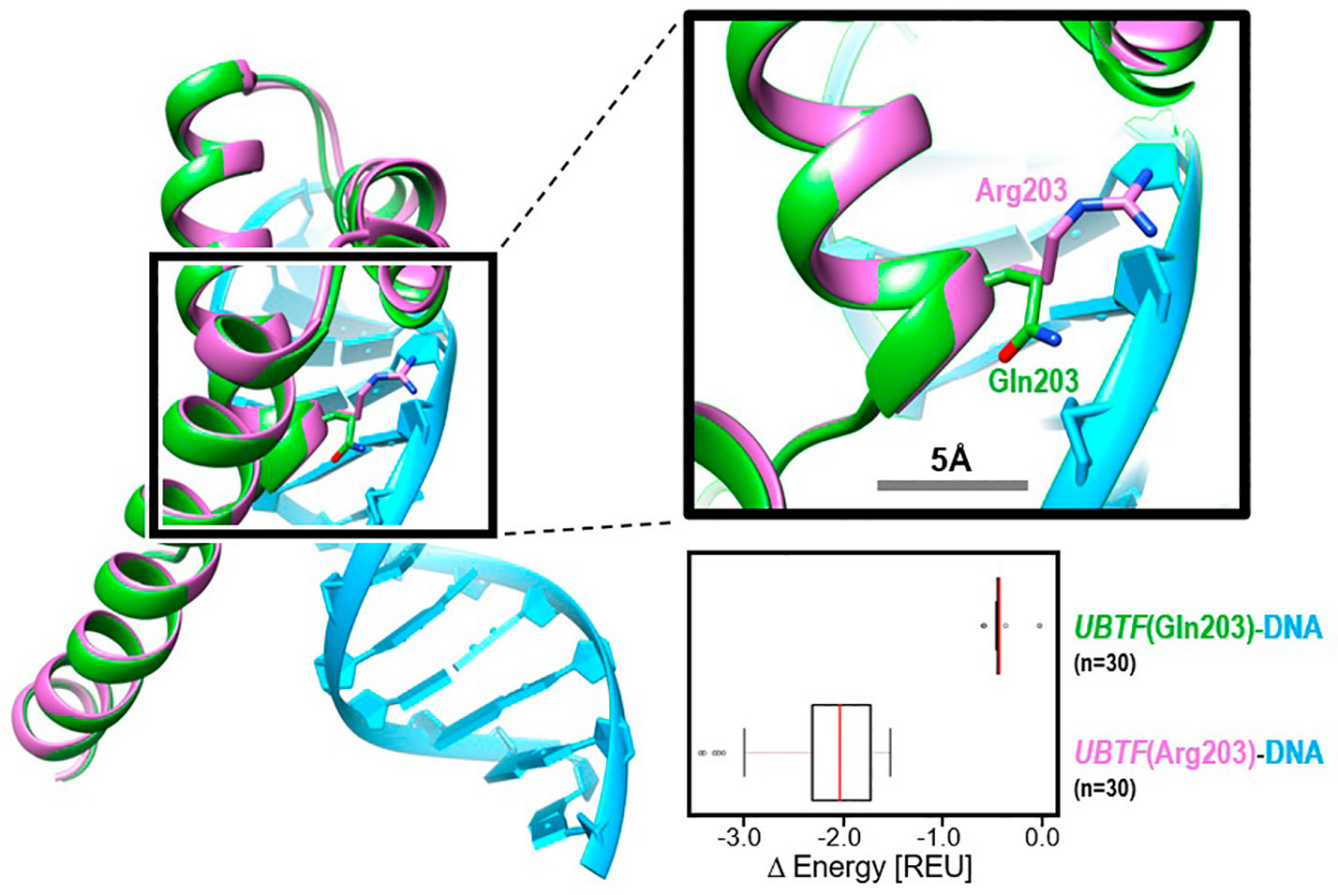
Model of second *UBTF* HMG‐box domain complexed with DNA. *UBTF* containing the wild‐type sequence (Gln203; green) is superimposed with that containing the proband's variant ((Gln203Arg); purple). Each *UBTF*‐DNA complex is shown in its lowest energy state. Detail shows predicted sidechain orientations, and their placement with respect to the DNA backbone (light blue). The boxplot summarizes the predicted energy differences for each of the 60 energy minimized models, with energy difference reported relative to the most energetically unfavorable model. The results show that *UBTF* (Arg203)‐DNA complexes were significantly more energetically stable than wild‐type (median energy difference of 1.6 REU; Mann–Whitney *p* = 1.545e‐11)

To better understand the effects p.(Gln203Arg) on the HMG box homology domain compared to the wild‐type residue, computational structural modeling was performed. The results show that *UBTF* p.(Gln203Arg)‐DNA complexes were significantly more energetically stable than those of the wild‐type (median energy difference of 1.6 REU; Mann–Whitney *p* = 1.545e‐11). This provides computational evidence that the p.(Gln203Arg) variant has an activating gain‐of‐function effect on the *UBTF* protein, UBF, with its more stable interaction with DNA possibly exaggerating its regulatory functions (Figure 1). Complete details of the modeling process are included in the supplement.

## 3 DISCUSSION

Recently, a de novo, pathogenic, gain‐of‐function, missense variant in *UBTF*, p.Glu210Lys, has been recurrently associated with the emerging neurodegenerative disorder CONDBA (Bastos et al., 2020; Edvardson et al., 2017; Ikeda et al., 2021; Sedláčková et al., 2019; Toro et al., 2018). Here, we report a CONDBA‐like proband with a unique, presumed de novo, missense *UBTF* variant, p.(Gln203Arg), who has additional, distinct phenotypic features of early‐life developmental delay and multiple neuroimaging abnormalities. These features differ from previous reports of delayed developmental regression and atrophy limited to the cerebellum and cerebrum in individuals with CONDBA. In our updated WES analysis, we present a comprehensive clinical and molecular assessment that led us to reclassify the *UBTF* p.(Gln203Arg) variant as likely pathogenic.

To date, *UBTF* p.(Gln203Arg) has not been reported in the literature. Currently, ClinVar does not contain an entry for this variant, and it has not been reported in the population database gnomAD. To test our hypothesis that p.Gln203Arg results in an activating gain‐of‐function effect on the *UBTF* protein, UBF, we constructed a computational model of both wild‐type *UBTF* and the p.(Gln203Arg) variant in complex with DNA. These simulations consistently predicted that *UBTF* p.(Gln203Arg) would form a more energetically stable *UBTF*‐DNA complex than wild‐type *UBTF*. Therefore, p.(Gln203Arg) likely results in a gain‐of‐function effect, increasing gene expression in our proband and providing a candidate mechanism for our syndrome. Further in vitro *and* in vivo studies are required to fully characterize the biological effects of this variant.

The increased diagnostic yield of WES in neurodevelopmental disease in comparison to other genetic techniques is well established (100,000 Genomes Project Pilot Investigators, 2021). However, our report illustrates the importance of WES interpretation in the context of a comprehensive clinical assessment with direct communication between laboratory and clinical teams. It also emphasizes the importance of trio versus proband‐only testing to pinpoint confirmed de novo variants. Although some neurodevelopmental disorders can be identified with traditional genetic approaches, the lower diagnostic yield may result in a stressful medical odyssey for patients and their families (Manickam et al., 2021).

The uniqueness of our proband's phenotype, combined with the distinct disease‐associated likely pathogenic variant detected, raise the possibility that we are reporting a novel syndrome. Alternatively, these findings may suggest that CONDBA can be caused by multiple variants in the *UBTF* gene with a broader phenotypic developmental spectrum than previously reported. Additional studies are required to further our understanding of this rare but devastating neurodevelopmental syndrome.

## AUTHOR CONTRIBUTIONS

*Study conception and design*: Rory J. Tinker, Tiffany Guess, Daniel Lubarsky, Binu Porath, Ping Mayo, Emily Solem, Laura A. Lee. Clinical care: Jennifer Brault, Mackenzie Mosera. *Analysis and interpretation of results*: Rory J. Tinker, Tiffany Guess, Daniel Lubarsky, Binu Porath, Mackenzie Mosera, Ping Mayo, Emily Solem, Laura A. Lee, Asha Sharam, Jennifer Brault. *Computational modeling*: David C. Rinker, Jonathan H. Sheehan. *Draft manuscript preparation*: Rory J. Tinker, Tiffany Guess, Daniel Lubarsky, Jennifer Brault. All authors reviewed the results and approved the final version of the manuscript.

## FUNDING INFORMATION

None.

## CONFLICT OF INTEREST

None.

## DECLARATIONS

RJT has provided paid consulting services to Sofinnova Partners.

## ETHICS APPROVAL

No IRB approval required for isolated case reports as per VUMC guidelines. All authors approved of the final submitted version.

## Supporting information


Supinfo S1

**Figure S1** Sagittal T1‐weighted MR image obtained at 18 months of age (a) demonstrates pontine hypoplasia (arrow). The corpus callosum appeared slightly thinner than on the MRI obtained at 12 months of age (not pictured). Coronal T2‐weighted MR images obtained at 12 months (b) and 18 months (c) of age show subtly increasing prominence of the cerebral sulci and cerebellar fissures, consistent with subtle cerebral and cerebellar volume loss or less than expected growth (the extra‐axial spaces usually become less prominent between 12 and 18 months of age). Comparison between axial T2‐weighted images obtained at 12 (d) and 18 (e) months of age shows slightly increasing ventricular caliber (further evidence of subtle volume loss) and evidence of hypomyelination. On the examination obtained at 18 months of age, there is no appreciable increase in T2 hypointensity of the cerebral white matter. In addition, there is less than expected difference in signal between gray and white matter compared to normal individuals of the same age. Axial T2 FLAIR obtained at 18 months of age (f) shows abnormally hyperintense cerebral white matter signal (for age), compatible with hypomyelination, as well as volume loss and abnormal T2 prolongation of the thalami (arrows)

**Figure S2** (a) Illustration of the *UBTF* gene structure and localization of both the novel, likely pathogenic variant c.608A>G (p.Gln203Arg) identified in the proband and the recurrent pathogenic variant c.628G>A (p.Glu210Lys) in exon 7 (NM_014233.3). (b) Electropherogram of sequencing results from the proband and his parents showing the de novo A>G substitution at nucleotide position 608

**Table S1** Phenotype of the proband compared to that of other CONDBA individuals previously reported in the literature

## Data Availability

The data that support the findings of this study are available from the corresponding author upon reasonable request.
