# Correction to “A novel, likely pathogenic variant in *UBTF*‐related neurodegeneration with brain atrophy is associated with a severe divergent neurodevelopmental phenotype”

**DOI:** 10.1002/mgg3.2189

**Published:** 2023-05-08

Tinker, R. J., Guess, T., Rinker, D. C., Sheehan, J. H., Lubarsky, D., Porath, B., Mosera, M., Mayo, P., Solem, E., Lee, L. A., Sarma, A., & Brault, J. (2022). A novel, likely pathogenic variant in *UBTF*‐related neurodegeneration with brain atrophy is associated with a severe divergent neurodevelopmental phenotype. *Molecular Genetics & Genomic Medicine*, 10, e2054. https://doi.org/10.1002/mgg3.2054

In the originally pubished article, author A. Sarma's name was misspelled as A. Sharam. This has been updated in the online version of the article.

We apologize for this error.

