# Supplementary material for: A novel, likely pathogenic variant in UBTF‐related neurodegeneration with brain atrophy is associated with a severe divergent neurodevelopmental phenotype
Source: Mol Genet Genomic Med. 2022 Sep 15;10(12):e2054. doi: 10.1002/mgg3.2054 (PMC9747545; doi:10.1002/mgg3.2054)
Supplement: Supplementary file 1 — Supinfo S1 [file MGG3-10-e2054-s003.docx]

Supplementary data

Written informed consent was obtained for whole exome sequencing and publication of clinical findings.

**Whole Exome Sequencing**

Whole Exome Sequencing (WES) was initially performed on the proband only, after obtaining appropriate informed consent. Prior to sequencing, nucleic acid from the patient’s blood sample was enriched for coding and adjacent non-coding regions of the genes using the IDT X-Gen Exome (Integrated DNA Technologies, Coralville, IA). The library products were sequenced with 2 by 150 bp reads on the Illumina NovaSeq sequencing instrument (Illumina, San Diego, CA). After alignment to the reference genome (UCSC hg19), off target, low quality, and duplicate reads were removed from analysis, and variants were detected with several different variant calling algorithms, including ones developed in the Genosity Lab (Iselin, NJ). The targeted coding exons and splice junctions of the known protein-coding RefSeq genes were assessed for the average depth of coverage and other data quality thresholds. All sequence alterations are described according to the Human Genome Variation Society (HGVS) nomenclature guidelines and are classified according to the guidelines for sequence variant interpretation of the American College of Medical Genetics and Genomics (ACMG). Variant classification categories include pathogenic, likely pathogenic, variant of uncertain significance (VUS), likely benign, and benign with likely benign and benign variants excluded from the report.

The following filters were applied prior to analysis: removal of known artifacts, removal variants with an overall frequency of more than 1% in gnomAD (recessive variants of interest with higher gnomAD frequency subsequently analyzed), removal of synonymous and 5’ and 3’ UTR variants, and inclusion of exonic and splice-site variants. Synonymous and intronic variants within 10 base pairs from an intron-exon border are reviewed, but only variants within 5 bp of intron-exon borders are reported unless they fit the ACMG criteria for pathogenic or likely pathogenic ^10^

5’ and 3’ UTR variants are not reviewed unless they fit the patient’s phenotype, are rare (< 1% frequency in gnomAD), and/or occur near a start/stop junction. Additionally, filtering included the use of Human Phenotype Ontology (HPO)^7^. The HPO terms used in the case were: hypoplasia of the ventral pons (6850), staring gaze (25401), delayed gross motor development (2194), gastroesophageal reflux (2020), microcephaly (252), hypotonia (1252), global developmental delay (1263), feeding difficulties (11968), motor delay (1270), and failure to thrive (1508). All *de novo* variants are manually reviewed, as are any frameshift, stop gain, stop loss, or known deleterious variants even if they do not fall within the phenotype filter. Use of these filters yielded a total of 78 variants, of which five variants were identified as they had at least some phenotypic overlap with the proband’s phenotype and were rare variants (Supplementary Table 1.).

**Modeling the effect of *UBTF* p.Gln203Arg on the HMG box homology** **domain**The proband’s variant (p.Gln203Arg) occurs in the second HMG Box Domain of *UBTF* (amino acid positions 195-265). To construct a model the *UBTF* HMG box homology domain in complex with DNA, we began with the NMR structure from a homologous HMG-box domain from Sry (1j46), a structure where the HMG-box domain has been experimentally determined while complexed with DNA^18^. We next extracted the second *UBTF* HMG-box homology domain from the AlphaFold2 model^19^ for *UBTF* (amino acid positions 195-265) and superimposed the *UBTF* domain with the Sry HMG-box domain in 1j46 (Chimera Matchmaker). The *UBTF* HMG box homology domain superimposes over the Sry HMG box homology domain with very low (1.089 Å) root mean square deviation. We then removed the Sry HMG box homology domain from the model. Finally, the *UBTF*-DNA complex was refined using the Rosetta software suite^20^ (Rosetta v.3.12) to carry out a constrained relax of the model in the Rosetta force field (Rosetta Energy Function 2015), followed by 20 independent, unconstrained relaxations to minimize free energy of the structure. Energy scores of the 20 models (expressed in terms of Rosetta Energy Units (REU), where 1 REU is roughly comparable to 1-3 kcal/mole) all converged to similar values (average= -219.828 REU; s.d.= 0.039 REU). The lowest energy model was selected from these 20 to carry forward into the comparative simulations.

To estimate the energetic consequences of the Gln203Arg substitution, we then allowed Rosetta to rebuild the residue sidechain at position 203 as either a glutamine (wildtype) or arginine (proband’s variant), followed by iterations of unconstrained relaxations. This process was independently carried out 30 times for each of the two protein variants (60 runs total). Rosetta energy scores were then gathered for the 60 runs, minimized *UBTF*-DNA complexes, and changes in overall free energy calculated by normalization to highest scoring (most energetically unstable) structure. Python was used to statistically analyze (SciPy) and visualize (matplotlib) the simulation results.

Supplementary Figure Captions
**Figure 1.** Sagittal T1-weighted MR image obtained at 18 months of age **(A)** demonstrates pontine hypoplasia (arrow). The corpus callosum appeared slightly thinner than on the MRI obtained at 12 months of age (not pictured). Coronal T2-weighted MR images obtained at 12 months **(B)** and 18 months **(C)** of age show subtly increasing prominence of the cerebral sulci and cerebellar fissures, consistent with subtle cerebral and cerebellar volume loss or less than expected growth (the extra-axial spaces usually become less prominent between 12 and 18 months of age). Comparison between axial T2-weighted images obtained at 12 **(D)** and 18 **(E)** months of age shows slightly increasing ventricular caliber (further evidence of subtle volume loss) and evidence of hypomyelination. On the examination obtained at 18 months of age, there is no appreciable increase in T2 hypointensity of the cerebral white matter. In addition, there is less than expected difference in signal between gray and white matter compared to normal individuals of the same age. Axial T2 FLAIR obtained at 18 months of age **(F)** shows abnormally hyperintense cerebral white matter signal (for age), compatible with hypomyelination, as well as volume loss and abnormal T2 prolongation of the thalami (arrows).

**Figure 2.** **(A)** Illustration of the *UBTF* gene structure and localization of both the novel, likely pathogenic variant c.608A>G (p.Gln203Arg) identified in the proband and the recurrent pathogenic variant c.628G>A (p.Glu210Lys) in exon 7 (NM_014233.3). **(B)** Electropherogram of sequencing results from the proband and his parents showing the de novo A>G substitution at nucleotide position 608
