# Supplementary figures and images for: A novel, likely pathogenic variant in UBTF‐related neurodegeneration with brain atrophy is associated with a severe divergent neurodevelopmental phenotype
Source: Mol Genet Genomic Med. 2022 Sep 15;10(12):e2054. doi: 10.1002/mgg3.2054 (PMC9747545; doi:10.1002/mgg3.2054)

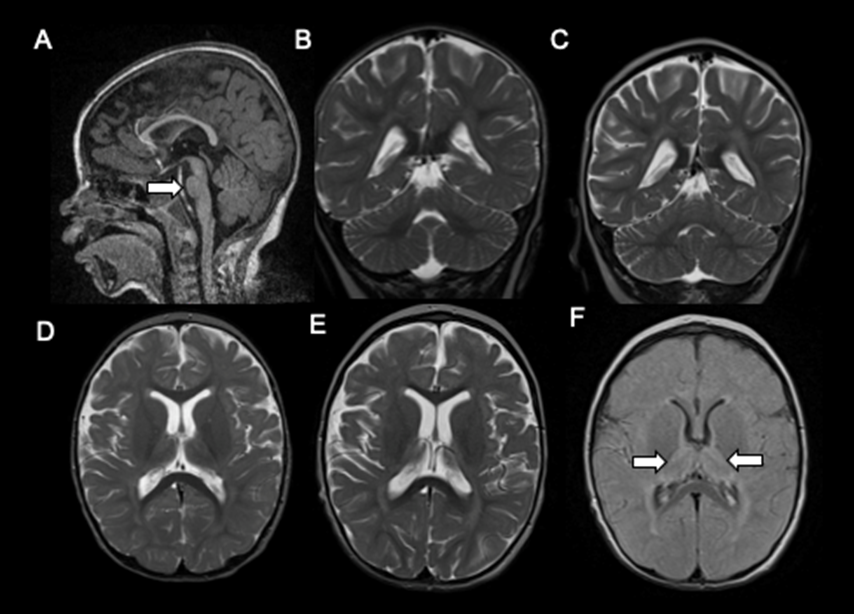

Supplement: Supplementary file 2 — Figure S1 Sagittal T1‐weighted MR image obtained at 18 months of age (a) demonstrates pontine hypoplasia (arrow). The corpus callosum appeared slightly thinner than on the MRI obtained at 12 months of age (not pictured). Coronal T2‐weighted MR images obtained at 12 months (b) and 18 months (c) of age show subtly increasing prominence of the cerebral sulci and cerebellar fissures, consistent with subtle cerebral and cerebellar volume loss or less than expected growth (the extra‐axial spaces usually become less prominent between 12 and 18 months of age). Comparison between axial T2‐weighted images obtained at 12 (d) and 18 (e) months of age shows slightly increasing ventricular caliber (further evidence of subtle volume loss) and evidence of hypomyelination. On the examination obtained at 18 months of age, there is no appreciable increase in T2 hypointensity of the cerebral white matter. In addition, there is less than expected difference in signal between gray and white matter compared to normal individuals of the same age. Axial T2 FLAIR obtained at 18 months of age (f) shows abnormally hyperintense cerebral white matter signal (for age), compatible with hypomyelination, as well as volume loss and abnormal T2 prolongation of the thalami (arrows) [file MGG3-10-e2054-s004.jpeg]

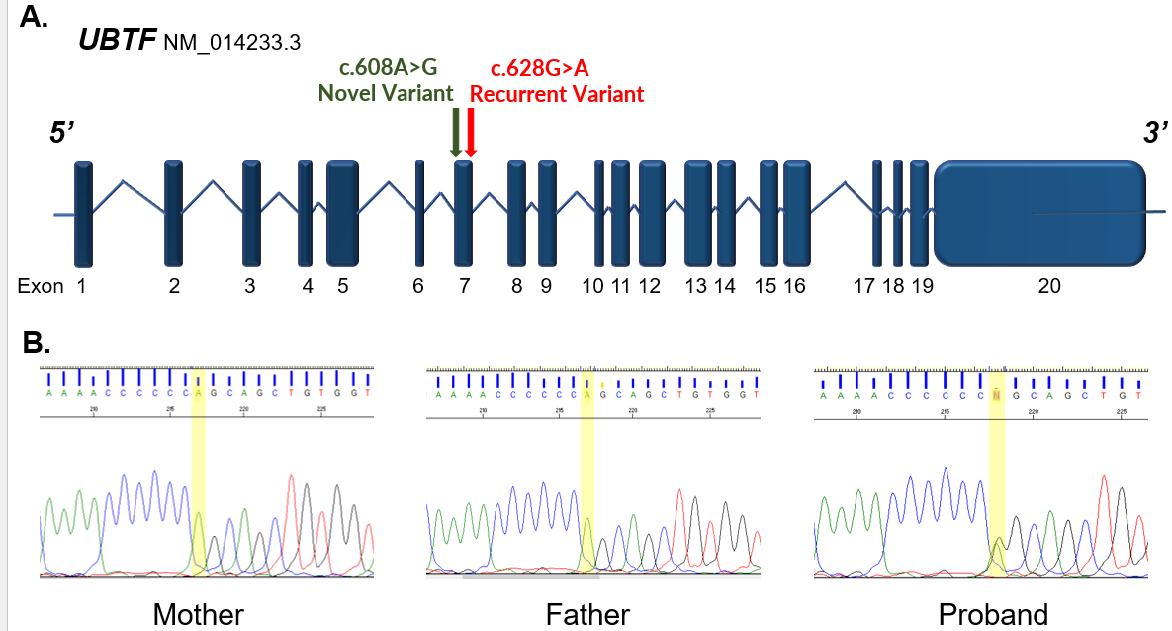

Supplement: Supplementary file 3 — Figure S2 (a) Illustration of the UBTF gene structure and localization of both the novel, likely pathogenic variant c.608A>G (p.Gln203Arg) identified in the proband and the recurrent pathogenic variant c.628G>A (p.Glu210Lys) in exon 7 (NM_014233.3). (b) Electropherogram of sequencing results from the proband and his parents showing the de novo A>G substitution at nucleotide position 608 [file MGG3-10-e2054-s002.jpeg]
