# Supplementary material for: A novel, likely pathogenic variant in UBTF‐related neurodegeneration with brain atrophy is associated with a severe divergent neurodevelopmental phenotype
Source: Mol Genet Genomic Med. 2022 Sep 15;10(12):e2054. doi: 10.1002/mgg3.2054 (PMC9747545; doi:10.1002/mgg3.2054)
Supplement: Supplementary file 4 — Table S1 Phenotype of the proband compared to that of other CONDBA individuals previously reported in the literature [file MGG3-10-e2054-s001.docx]

Supplementary Tables
Supplementary Table 1. Phenotype of the proband compared to that of other CONDBA individuals previously reported in the literature.

| Phenotypic features | Reported literature | Current proband |
| --- | --- | --- |
| Demographic | | |
| Sex | 5M 9F | F |
| Developmental phenotype | | |
| Developmental delay | 6/14 mild single domain delay in the first 2 years of life | Severe global developmental delay resulting in developmental stalling 9 months of age |
| Age at regression | Present in all 14 cases Median 3 years  Mean 3.55 Range 2 to 7 years | Mild developmental regression from poor developmental baseline  at 18 months of age consisting of the loss of the ability to swallow |
| Neurocognitive phenotype | Progressive intellectual disability  14/14 | Not present at 19 months of age but low developmental baseline |
| Neurological phenotype | | |
| Microcephaly | 7/14 | Yes |
| Ataxia | 9/14 | Yes |
| Extrapyramidal signs | 12/14 | Yes |
| Pyramidal signs | 13/14 | Yes |
| Early onset parkinsonism | 1/14 | No |
| Seizures | 5/14 | Epileptiform discharges on EEG No seizures to date |
| Neurological investigations | | |
| Brain MRI | 14/14 cerebral and white matter atrophy  10/13 cerebellar atrophy | Progressive cerebral and cerebellar volume loss Abnormal T2 prolongation in the periventricular white matter and thalami  Callosal thinning  Small ventral pons |
| EEG | 4 abnormal (no details) 1 abnormal background rhythm 1 diffuse β activity with sharp waves 1 continuous bilateral FT spikes with a slow background 1 transient pattern of semi-periodic slow waves 1 low-voltage fast activity without clinical correlate 5 normal | Frequent left fronto-polar maximal sharp wave consistent with a potential epileptogenicity in the left fronto-polar region. |

Supplementary table 2

| Gene | **Variant Details** | **Read Depth** | **Zygosity, Inheritance** | **gnomAD frequency** | **Predicted Pathogenicity** | **ACMG Criteria** |  |
| --- | --- | --- | --- | --- | --- | --- | --- |
| *UBTF* | NM014233.3: c.608A>G, p.(Gln203)Arg | 63 | Heterozygous, *De novo* | 0.000000% | Likely pathogenic | PS2, PM2, PP2, PP3 |  |
|  |  |  |  |  |  |  |  |
|  |  |  |  |  |  |  |  |
| *SETD5* | NM_001080517.2: c.2023A>G, p.Thr675Ala | 66 | Heterozygous, Paternally inherited | 0.001206% | Variant of Uncertain Significance | PM2, BP4 |  |
|  |  |  |  |  |  |  |  |
|  |  |  |  |  |  |  |  |
| *LRPPRC* | NM_133259.3: c.2753C>T, p.Ala918Val | 31 | Heterozygous, * | 0.001062% | Variant of Uncertain Significance | PM2 |  |
|  |  |  |  |  |  |  |  |
|  |  |  |  |  |  |  |  |
| *POMK* | NM_032237.4: c.137G>A, p.Arg46Gln | 54 | Heterozygous, * | 0.000000% | Variant of Uncertain Significance | PM2, BP4 |  |
|  |  |  |  |  |  |  |  |
|  |  |  |  |  |  |  |  |
| *WASHC4* | NM_015275.2: c.1704A>C, p.Gln568His | 66 | Heterozygous, * | 0.001068% | Variant of Uncertain Significance | PM2 |  |
|  |  |  |  |  |  |  |  |
|  |  |  |  |  |  |  |  |

* Inheritance unknown
WES indented VUS at 15 months of age
